# Data for the cytotoxicity, self-assembling properties and synthesis of 4-pyridinium-1,4-dihydropyridines

**DOI:** 10.1016/j.dib.2020.106545

**Published:** 2020-11-19

**Authors:** Klavs Pajuste, Martins Rucins, Ilona Domracheva, Arkadij Sobolev, Nadiia Pikun, Mara Plotniece, Gunars Duburs, Karlis Pajuste, Aiva Plotniece

**Affiliations:** a Latvian Institute of Organic Synthesis, Aizkraukles str. 21, LV-1006, Riga, Latvia; b Department of Pharmaceutical Chemistry, Faculty of Pharmacy, Riga Stradiņš University, Dzirciema str. 16, LV-1007, Riga, Latvia

**Keywords:** 1,4-dihydropyridine, Pyridinium, Quaternisation, Cytotoxicity, Self-assembling, Nanoparticles, Structure-activity relationships

## Abstract

In this data file the synthetic procedures for preparation of the original 4-pyridinium-1,4-dihydropyridines (4-Py-1,4-DHP) and their parent compounds – dialkyl 2,6-dimethyl-4-(3-pyridyl)-1,4-dihydropyridine-3,5-dicarboxylates were described. In total, 5 unpublished compounds were obtained and characterised. All the structures of original compounds were confirmed by Nuclear Magnetic Resonance (NMR, including ^1^H NMR and ^13^C NMR) and low resolution mass spectra (MS) data. Additionally, the cytotoxic properties of four 4-Py-1,4-DHPs were evaluated on 3 cell lines – normal NIH3T3 (mouse embryonic fibroblast), cancerous HT-1080 (human lung fibrosarcoma) and MH-22A (mouse hepatoma) and self-assembling properties were studied and characterisation of formed nanoparticles were performed using dynamic light scattering technique. In this article provided data are directly related to the previously published research articles – “Novel cationic amphiphilic 1,4-dihydropyridine derivatives for DNA delivery” [1] where compound 5 was tested as gene delivery agent without full physico-chemical characterisation and “Synthesis and studies of calcium channel blocking and antioxidant activities of novel 4-pyridinium and/or N-propargyl substituted 1,4-dihydropyridine derivatives” [2] where synthesis and physico-chemical characterisation as well as calcium channel blocking and antioxidant activities were described for compound 6. Synthesis of other compounds – parent 1,4-DHPs 1 and 2, and 4-Py-1,4-DHPs 3–5, their characterisation, estimation of cytotoxicity and self-assembling properties for all 4-Py-1,4-DHPs 3–6 are reported herein for the first time. Information provided in this data file can be used in medicinal chemistry by other scientists to estimate structure-activity relationships for the analysis and construction of various cationic 1,4-dihydropyridine derivatives and related heterocycles.

## Specifications Table

**Table:** 

Subject	Medicinal chemistry
Specific subject area	Organic chemistry, quaternisation, cytotoxicity, self-assembling, nanoparticles
Type of data	Synthetic scheme, general protocol for synthesis, table with structures and cytotoxicity and calculated basal toxicity data, table with characteristic parameters of nanoparticles, NMR data; in supplementary data – NMR spectra, LC-MS data.
How data were acquired	^1^H and ^13^C NMR spectra were recorded at 400 MHz (^1^H) and 100 MHz (^13^C) operating frequencies with a Bruker Avance Neo 400 MHz. Multiplicities are abbreviated as: s, singlet; d, doublet; t, triplet; q, quartet; m, multiplet; dd, double doublet; dt, double of triplets; ddd, double doublet of doublets; br.s, broad singlet. Chemical shifts are presented in parts per million (ppm) and referred to the residual signals of the non-deuterated CDCl_3_ (*δ*: 7.26) for ^1^H NMR spectra and CDCl_3_ (*δ*: 77.0) for ^13^C NMR, respectively. Coupling constants *J* were reported in hertz (Hz).
	Elemental Combustion System ECS 4010 (Costech Instruments) was used to determine elemental analyses.
	Low resolution mass spectra (MS) were determined on an Acquity UPLC system (Waters) connected to a Waters SQ Detector-2 operating in the ESI positive ion mode on a Waters Acquity UPLC® BEH C18 column (1.7 µm, 2.1 × 50 mm, using gradient elution with acetonitrile (0.01% formic acid) in water (0.01% formic acid). The purities of the compounds were analysed by HPLC on Waters Alliance 2695 system and Waters 2485 UV/Vis detector equipped with SymmetryShield RP18 column (5 µm, 4.6 × 150 mm) using a gradient elution with methanol/water (v/v), at a flow rate of 1 mL/min. Peak areas were determined electronically with Waters Empower 2 chromatography data system.
	Dynamic light scattering (DLS) technique (Zetasizer Nano ZSP (Malvern Panalytical Ltd.) instrument with Malvern Instruments Ltd. Software 7.12) was used for evaluation of self-assembling properties of final 4-pyridinium-1,4-DHP derivatives and characterisation of obtained nanoparticles.
Data format	Raw and analysed
Parameters for data collection	Data were collected for characterisation purposes.
Description of data collection	Data were collected via the raw output files from the respective hardware. ^1^H and ^13^C NMR spectra were recorded as fid files.
Data source location	Latvian Institute of Organic Synthesis, Riga, Latvia
Data accessibility	Data are provided within the article and supplementary materials.
Related research article	Rucins M.; Kaldre D.; Pajuste K.; Fernandes M.A.S.; Vicente J.A.F.; Klimaviciusa L.; Jaschenko E.; Kanepe-Lapsa I.; Shestakova I.; Plotniece M.; Gosteva M.; Sobolev A.; Jansone B.; Muceniece R.; Klusa V.; Plotniece A. Synthesis and studies of calcium channel blocking and antioxidant activities of novel 4-pyridinium and/or N-propargyl substituted 1,4-dihydropyridine derivatives. *C. R. Chimie*, 2014, 17, 69–80. http://dx.doi.org/10.1016/j.crci.2013.07.003
	Hyvönen Z.; Plotniece A.; Reine I.; Chekavichus B.; Duburs G.; Urtti A. Novel cationic amphiphilic 1,4-dihydropyridine derivatives for DNA delivery. *Biochim. Biophys. Acta*, 2000, 1509, 451–466. 10.1016/S0005–2736(00)00,327–8

## Value of the Data

• The data contain the synthetic procedures for preparation of dialkyl 2,6-dimethyl-4-(3-pyridyl)-1,4-dihydropyridine-3,5-dicarboxylates with following quaternisation of pyridine moiety giving 4-pyridinium-1,4-dihydropyridines which may serve as valuable guidance for other organic chemists.
• The data provide characterisation of physico-chemical properties of original compounds – parent 4-pyridine-1,4-DHPs and quaternised pyridine moiety containing 1,4-DHPs which have not been reported before.
• Moreover, the described synthetic procedures and obtained spectral data will be useful for preparation and structure elucidation of representatives in related heterocycles.
• Besides the estimated cytotoxicity and basal toxicity data of quaternised pyridine moiety containing 1,4-DHPs may be used for studies and definitions of structure-activity relationships.
• Additionally, self-assembling properties of quaternised pyridine moiety containing 1,4-DHPs were estimated and obtained nanoparticles characterised by dynamic light scattering measurements. These data may be respected for the design and development of delivery systems.

## 1 Data Description

**Fig. 1:**
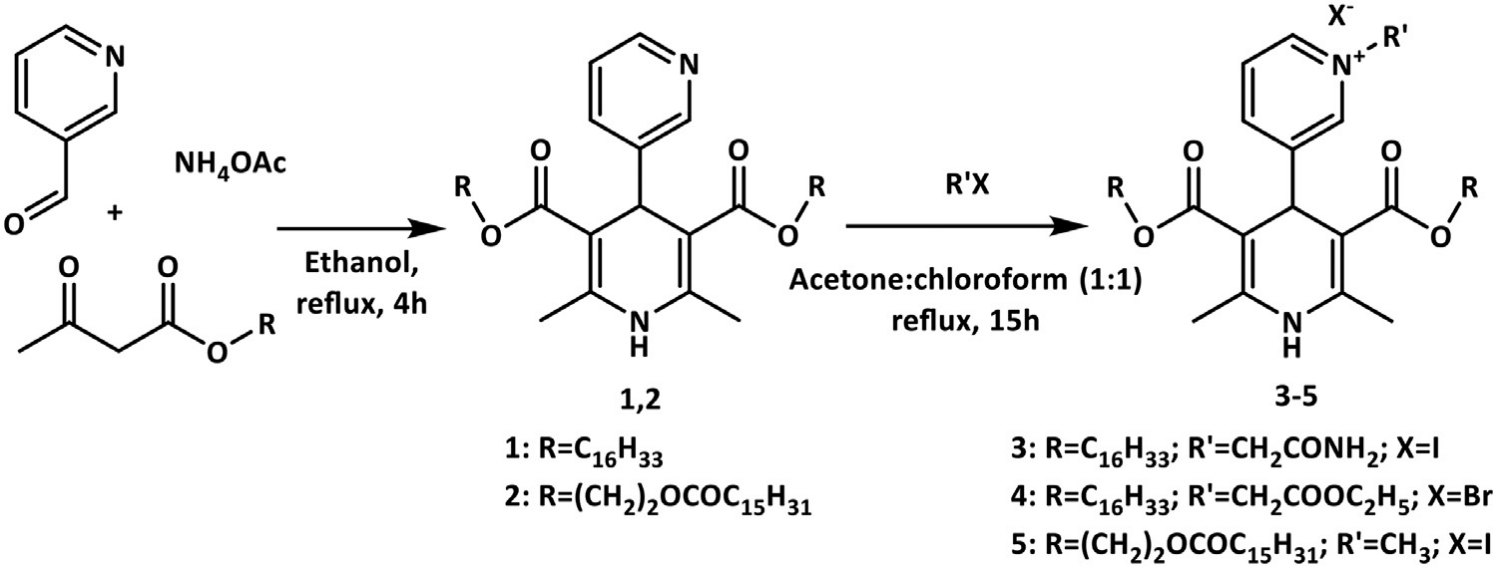
Synthesis of 4-pyridyl-1,4-DHPs 1,2 and 4-N-pyridinium-1,4-DHPs 3–5.

As a part of our research topic towards the development of novel pyridinium moieties containing biologically active compounds, we continue studies on original single-charged cationic lipid-like compounds on the 1,4-DHP core. Synthetic procedure and the structures of 1,4-DHP derivatives with quaternised pyridine moiety at the position 4 were depicted in Fig. 1.

According to the literature data 4-phenyl-, 4-pyridyl- and 4-unsubstituted 1,4-DHPs is performed *via* the classical well-known synthetic procedures and target compounds can be obtained in good yields (78–80%) [3], [4]. Also in this case, the parent 4-pyridyl-1,4-DHPs 1 and 2 as intermediates for preparation of target cationic moiety containing 1,4-DHPs were synthesised with high yields, 71% and 75% respectively, *via* typical Hantzsch method performing the one-pot cyclocondensation of the corresponding acetoacetate, 3-pyridinecarboxaldehyde and ammonium acetate in ethanol under refluxing.

The preparation of 4-pyridinium-1,4-DHPs 3–5 were performed by quaternisation of pyridine moiety of the 4-pyridyl-1,4-DHPs 1 or 2 with the corresponding halides in acetone:chloroform mixture under reflux. [5], [6] Reaction times and yields of the product are dependant on the structure of the alkyl halide. An excess of the alkylation agent was used to reduce the reaction time [7]. All 4-pyridinium-1,4-DHPs 3–5 were synthesised with high yields: 71–85%.

Structures of original compounds – parent 4-pyridyl-1,4-DHPs 1 and 2, and 4-(N-alkyl)pyridinium-1,4-DHPs 3–5, their characterisation are reported herein for the first time and confirmed by ^1^H and ^13^C NMR spectra, MS and elemental analysis data (Spectra see in the Supplementary material).

^1^H NMR spectrum and elemental analysis data of diethyl 4-(1-hexadecylpyridin-1-ium-3-yl)-2,6-dimethyl-1,4-dihydropyridine-3,5-dicarboxylate bromide (6) were in agreement with the previously reported [2].

The purities of the studied compounds were at least 97% according to high-performance liquid chromatography (HPLC) data.

Previously it was shown that related compound – 4-(N-dodecyl)pyridinium-1,4-DHPs demonstrated the ability to block brain calcium channels and improve memory by enhancing the GABAergic processes [8].

Compound 6 was added to the set of chosen 1,4-DHPs 3–5 for further evaluation of cytotoxicity and self-assembling properties as well as estimation of structure-activity relationships. 4-Py-1,4-DHP 6 containing N-hexadecyl pyridinium moiety at position 4 and ethyl ester moieties at positions 3 and 5 of the 1,4-DHP cycle in the opposite other 4-Py-1,4-DHPs 3–5 which possessed short alkyl or acyl substituent in pyridinium moiety at position 4 of the 1,4-DHP cycle and long, lipid-like ester moieties at positions 3 and 5 of the 1,4-DHP cycle.

The evaluation of cytotoxicity for all 4-Py-1,4-DHPs 3–6 *in vitro* was assessed using the MTT {3-(4,5-dimethylthiazol-2-yl)-2,5-diphenyltetrazolinium bromide} and CV {tris(4-(dimethylamino)phenyl)methylium chloride} assays on two monolayer tumour cell lines, namely HT-1080 (human fibrosarcoma) and MH-22A (mouse hepatoma) and also the compound influence on “normal” mouse fibroblasts (NIH3T3) was estimated for the studies of structure–activity relationships. Using an alternative *in vitro* method it is possible to estimate possible toxicity of new compounds and selected compounds for further study that vastly reducing the number of animal experiments. The results are presented in Table 1.

**Table 1:** Cytotoxicity and calculated basal toxicity of 4-pyridinium-1,4-dihydropyridines 3–6 .

Comp.	R	R’	X^−^	HT-1080		MH-22A		NIH3T3	
				IC_50_ (CV)	IC_50_ (MTT)	IC_50_ (CV)	IC_50_ (MTT)	IC_50_ (NR)	LD_50_
				µg/mL	µg/mL	µg/mL	µg/mL	µg/mL	mg/kg
3	C_16_H_33_	CH_2_CONH_2_	I	70 ± 11	26 ± 5	100 ± 16	32 ± 8	16 ± 2	638
4	C_16_H_33_	CH_2_COOC_2_H_5_	Br	*	*	*	*	*	>2000
5	(CH_2_)_2_OCOC_15_H_31_	CH_3_	I	12 ± 2	20 ± 8	10 ± 2	12 ± 3	40 ± 8	1020
6	C_2_H_5_	C_16_H_33_	Br	<<1	<<1	<<1	<<1	4 ± 0.4	295 ± 45

* – not detected.

IC_50_ is a quantitative measure of the compound concentration (µg/ml), at which 50% of the cells die. CV is a triarylmethane dye that can bind to ribose type molecules such as DNA in nuclei. CV staining can be used to quantify the total DNA of the remaining population and thus is used to determine the number of live cells based on the concentration of the dye which remains after staining. MTT is a standard colorimetric assay used to measure a cellular proliferation. Yellow MTT is reduced to purple formazan in the mitochondria of living cells.

The estimated LD_50_ values were calculated and obtained results in accordance with 4 toxicity categories (see paragraph 2.4.3.) showed that diethyl 4-(1-hexadecylpyridin-1-ium-3-yl)−2,6-dimethyl-1,4-dihydropyridine-3,5-dicarboxylate bromide (comp. 6) were identified as slightly toxic (category 3) due to LD_50_ value 295 mg/kg. While other 4-pyridinium-1,4-DHPs 3–5 are practically non-toxic (category 4) with LD_50_ values 638, >2000 and 1020 mg/kg, respectively.

Dynamic light scattering (DLS) technique was used for evaluation of self-assembling properties of 4-pyridinium-1,4-DHP derivatives 3–6 and characterisation of obtained nanoparticles, including determination of average diameter (D_av_), zeta-potential (Zeta-pot.) and polydispersity index (PDI). Samples were prepared by the thin-film hydration method. The results are obtained for freshly prepared samples after 24 h storage and their values are presented in Table 2.

**Table 2:** Values of average diameter (D_av_), zeta-potential (Zeta-pot.) and polydispersity index (PDI) of nanoparticles formed by 4-pyridinium-1,4-DHP derivatives 3–6 obtained by dynamic light scattering (DLS) measurements. The average diameter (D_av_) depicts the average hydrodynamic diameter of nanoparticles in the tested sample; the PDI value describes polydispersity of the sample; the zeta-potential gives information about the surface charge of nanoparticles.

Comp.	D_av_	PDI	Zeta-pot.
3	*	1	*
4	*	1	*
5	88 ± 3	0.346 ± 0.034	35.1 ± 3.4
6	585 ± 20	0.334 ± 0.153	0.39 ± 0.54

* – not detected.

It was demonstrated that 4-pyridinium-1,4-DHPs 5 and 6 formed relatively homogenous nanoparticles, their PDI values are around 0.340 and average particle diameter around 90 and 600 nm, respectively. While other 4-pyridinium-1,4-DHPs 3 and 4 formed very heterogeneous samples with PDI value 1, which means a broad particle size distribution and will not be discussed further here, because these compounds are not perspective for further studies.

## 2 Experimental Design, Materials and Methods

### 2.1 General information

All the necessary reagents and solvents were bought from commercial suppliers (ACROS, Sigma-Aldrich) and used without further purification. Progress of reactions was monitored with thin-layer chromatography (TLC) on Silica gel 60 F_254_ aluminium sheets 20 × 20 cm; eluent; EtOAc:hexane (1:5) for compound 1 and EtOAc:hexane (1:1) for compound 2 and EtOH:ammonia solution, 25% (5:1) for 4-Py-1,4-DHPs 3–6. Melting points were performed on an OptiMelt (SRS Stanford Research Systems).

### 2.2 General procedure for synthesis of 1,4-dihydropyridines 1 and 2

The 1,4-dihydropyridines 1 and 2 were synthesised from the corresponding esters of acetoacetic acid (2.0 eq) which were obtained according to [9] for compound 1 and [10] for compound 2, 3-pyridinecarboxaldehyde (1.0 eq) and ammonium acetate (1.2 eq) by refluxing of the reaction mixture in ethanol for 4 h. Then reaction mixture was cooled down to +4 °C, then precipitates were filtered off and recrystallised from hexane:ethanol (4:1).

#### 2.2.1 Dihexadecyl 2,6-dimethyl-4-(3-pyridyl)-1,4-dihydropyridine-3,5-dicarboxylate (1)



Yield: 71%. Light yellow powder; m.p. 138–140 °C; ^1^H NMR (400 MHz, CDCl_3_) δ: 8.53–8.50 (m, 1H), 8.36 (dd, 1H, *J* = 4.8 and 1.8 Hz), 7.59 (dt, 1H, *J* = 7.8 and 1.8 Hz), 7.14 (ddd, 1H, *J* = 7.8, 4.8 and 0.7 Hz), 5.92 (br.s, 1H), 4.98 (s, 1H), 4.07–3.97 (m, 4H), 2.34 (s, 6H), 1.63–1.54 (m, 4H), 1.26 (m, 52H), 0.90–0.85 (m, 6H) ppm. ^13^C NMR (100 MHz, CDCl_3_) δ: 167.34, 149.70, 147.40, 144.73, 143.33, 135.72, 123.18, 103.54, 64.29, 37.87, 32.08, 29.86, 29.83, 29.82, 29.78, 29.73, 29.52, 29.44, 28.84, 26.22, 22.84, 19.71, 14.27 ppm. Anal. Calcd. for C_46_H_78_N_2_O_4_: C%, 76.40; H%, 10.87; N%, 3.87. Found: C%, 76.40; H% 10.98; N% 3.93. MS (ESI+) *m/z* (relative intensity) 723 (([M]^+^*H*, 100%).

#### 2.2.2 Bis(2-hexadecanoyloxyethyl) 2,6-dimethyl-4-(3-pyridyl)-1,4-dihydropyridine-3,5-dicarboxylate (2)



Yield: 75%. White powder; m.p. 106–108 °C; ^1^H NMR (400 MHz, CDCl_3_) δ: 8.54–8.52 (m, 1H), 8.37 (dd, 1H, *J* = 4.8 and 1.8 Hz), 7.61 (dt, 1H, *J* = 7.9 and 1.8 Hz), 7.13 (ddd, 1H, *J* = 7.8, 4.8 and 0.7 Hz), 5.93 (s, 1H), 4.96 (s, 1H), 4.26–4.20 (m, 8H), 2.34 (s, 6H), 2.31–2.26 (m, 4H), 1.63–1.54 (m, 4H), 1.33–1.21 (m, 48H), 0.90–0.85 (m, 6H) ppm. ^13^C NMR (100 MHz, CDCl_3_) δ: 173.71, 166.78, 149.72, 147.50, 145.35, 143.15, 135.69, 123.13, 103.17, 62.10, 61.94, 37.83, 34.23, 32.07, 29.85, 29.82, 29.80, 29.79, 29.65, 29.51, 29.46, 29.31, 25.02, 22.83, 19.79, 14.26 ppm. Anal. Calcd. for C_50_H_82_N_2_O_8_: C%, 71.56; H%, 9.85; N%, 3.34. Found: C%, 71.56; H% 10.05; N% 3.42. MS (ESI+) *m/z* (relative intensity) 840 ([M]^+^*H*, 100%).

### 2.3 General procedure for synthesis of alkylated 1,4-dihydropyridines 3–5

The corresponding dialkyl 2′,6′-dimethyl-1′,4′-dihydro-[3,4′-*bipyridine*]-3′,5′-dicarboxylate 1 or 2 (1.0 eq) was dissolved in acetone:chloroform (1:1) mixture and 2-iodoacetamide (1.0 eq) or ethyl 2-bromoacetate (1.4 eq), or iodomethane (5.0 eq) was added and reaction mixture was refluxed for 15 h. Then reaction mixture was cooled down to +4 °C overnight, precipitates filtered off and recrystallised from ethanol.

#### 2.3.1 Dihexadecyl 4-[1-(2-*amino*-2-*oxo*-*ethyl*)*pyridin*-1-*ium*-3-*yl*]-2,6-dimethyl-1,4-dihydropyridine-3,5-dicarboxylate iodide (3)



Yield: 71%. Yellow powder; m.p. 140–142 °C; ^1^H NMR (400 MHz, CDCl_3_) δ: 9.00 (s, 1H), 8.80 (d, 1H, *J* = 6.1 Hz), 8.43 (d, 1H, *J* = 8.0 Hz), 7.90 (br.s, 1H), 7.79 (dd, 1H, *J* = 6.1 and 8.0 Hz), 7.16 (s, 1H), 6.26 (br.s, 1H), 5.92 (s, 2H), 5.11 (s, 1H), 4.02 (t, 4H, *J* = 6.8 Hz), 2.42 (s, 6H), 1.62–1.54 (m, 4H), 1.33–1.19 (m, 52H), 0.90–0.83 (m, 6H) ppm. ^13^C NMR (100 MHz, CDCl_3_) δ: 167.13, 165.55, 149.62, 147.36, 145.28, 144.89, 143.16, 125.96, 101.05, 64.85, 61.86, 39.38, 32.04, 29.83, 29.79, 29.78, 29.75, 29.70, 29.48, 29.42, 28.82, 26.24, 22.80, 20.22, 14.24 ppm. Anal. Calcd. for C_48_H_82_N_3_O_5_Br: C%, 63.49; H%, 9.38; N%, 4.63. Found: C%, 63.72; H% 9.38; N% 4.66. MS (ESI+) *m/z* (relative intensity) 780 ([M]^+^, 100%).

#### 2.3.2 Dihexadecyl 4-[1-(2-*ethoxy*-2-*oxo*-*ethyl*)*pyridin*-1-*ium*-3-*yl*]-2,6-dimethyl-1,4-dihydropyridine-3,5-dicarboxylate bromide (4)



Yield: 73%. Yellow powder; m.p. 98–100 °C; ^1^H NMR (400 MHz, CDCl_3_) δ: 9.26–9.19 (m, 1H), 8.77–8.70 (m, 1H), 8.45–8.41 (m, 1H), 8.39–8.32 (m, 1H), 7.86 (dd, 1H, *J* = 6.2 and 8.0 Hz), 6.00 (s, 2H), 5.12 (s, 1H), 4.26 (q, 2H, *J* = 7.2 Hz), 4.00 (t, 4H, *J* = 6.7 Hz), 2.47 (s, 6H), 1.60–1.50 (m, 4H), 1.28 (t, 3H, *J* = 7.2 Hz) overlap, 1.29–1.21 (m, 52H) overlap, 0.88–0.83 (m, 6H) ppm. ^13^C NMR (100 MHz, CDCl_3_) δ: 167.27, 165.45, 149.62, 148.40, 145.56, 144.88, 143.83, 126.91, 100.26, 64.68, 63.48, 61.10, 39.11, 32.01, 29.79, 29.75, 29.72, 29.65, 29.45, 29.38, 28.80, 26.20, 22.77, 19.66, 14.20, 14.11 ppm. Anal. Calcd. for C_50_H_85_N_2_O_6_Br: C%, 67.47; H%, 9.62; N%, 3.15. Found: C%, 67.39; H% 9.86; N% 3.09. MS (ESI+) *m/z* (relative intensity) 809 ([M]^+^, 100%).

#### 2.3.3 Bis(2-hexadecanoyloxyethyl) 2,6-dimethyl-4-(1-methylpyridin-1-ium-3-yl)-1,4-dihydropyridine-3,5-dicarboxylate iodide (5)



Yield: 85%. Yellow powder; m.p. 112–113 °C; ^1^H NMR (400 MHz, CDCl_3_) δ: 8.96 (s, 1H), 8.77 (d, 1H, *J* = 6.1 Hz), 8.48 (d, 1H, *J* = 8.1 Hz), 7.89 (dd, 1H, *J* = 8.1 and 6.1 Hz), 7.64 (s, 1H), 5.05 (s, 1H), 4.65 (s, 3H), 4.34–4.16 (m, 8H), 2.48 (s, 6H), 2.29 (t, 4H, *J* = 7.6 Hz), 1.64–1.55 (m, 4H), 1.35–1.20 (m, 48H), 0.91–0.84 (m, 6H) ppm. ^13^C NMR (100 MHz, CDCl_3_) δ: 173.77, 166.68, 149.51, 148.56, 144.92, 144.72, 142.27, 127.41, 100.26, 62.58, 61.84, 49.50, 38.94, 34.33, 32.06, 29.84, 29.80, 29.77, 29.64, 29.50, 29.44, 29.30, 25.07, 22.83, 20.20, 14.26 ppm. Anal. Calcd. for C_51_H_85_N_2_O_8_I: C%, 62.43; H%, 8.73; N%, 2.86. Found: C%, 62.13; H% 8.76; N% 2.85. MS (ESI+) *m/z* (relative intensity) 854 ([M]^+^, 100%).

### 2.4 Estimation of cytotoxicity

#### 2.4.1 Cell culture and measurement of cell viability

Tumour cell lines HT-1080 (Human connective tissue fibrosarcoma, ATCC® CCL-121™) and MH-22A (Mouse hepatosarcoma, ECACC, cat. Nr. 96,121,721) were used.

HT-1080 and MH-22A cells were seeded in 96-well plates in Dulbecco's modified Eagle's (DMEM) medium containing 10% foetal bovine serum, 4 mM l-Glutamine, w/o antibiotics and cultivated for 72 h by exposure to different concentrations of compounds. Cell viability was measured using 3-(4,5-dimethylthiazol-2-yl)-2,5-diphenyltetrazolinium bromide (MTT). In brief, after incubating with compounds, the culture medium was removed and fresh medium with 0.2 mg/mL MTT was added in each well of the plate. After incubation (3 h, 37 °C, 5% CO_2_) the medium with MTT was removed and 200 μL DMSO were added at once to each sample. The samples were tested at 540 nm on Tecan multiplate reader Infinite100. The IC_50_ was calculated using the program Graph Pad Prism® 3.0.

For the CV assay, cells were stained with 0.05% crystal violet (Sigma-Aldrich) in 30% methanol for 20 min at room temperature. After incubation the staining solution was removed, the cells were washed for 4 times with water. For dye solubilisation 200 μL a solution of 0.1 M citrate buffer, pH 4.2 with 50% ethanol; 1:1 v/v was added. The absorbence of the solution was measured using an Infinite M1000 Tecan microplate spectrophotometer at a wavelength of 570 nm [11].

#### 2.4.2 Basal cytotoxicity test

The Neutral Red Uptake (NRU) Assay was performed according to the standard protocol of Stokes [12] modified by NICEATM-ECVAM validation study [13]. The NRU cytotoxicity assay procedure based on the ability of viable cells to incorporate and bind neutral red, a supravital dye.

Balb/c NIH3T3 (Mouse Swiss Albino embryo fibroblast, ATCC® CRL-1658™) cells (9000 cells/well) were placed into 96-well plates for 24 h in Dulbecco's modified Eagle's (DMEM) medium containing 5% fetal bovine serum. Then exposed to the test compound over a range of eight concentrations (1000, 316, 100, 31, 10, 3, 1 μg/mL) for 24 h. Untreated cells were used as a control. After 24 h, the medium was removed from all plates. Then, 150 μL of neutral red solution was added (0.05 mg/mL NR in DMEM 24 h pre-incubated at 37 °C and then filtered before use through 0.22 µm syringe filter). Plates were incubated for 3 h and then cells were washed three times with PBS. The dye within viable cells was released by extraction with a mixture of acetic acid, ethanol and water (1:50:49). The absorbence of neutral red was measured using a spectrophotometer multiplate reader (TECAN, Infinite M1000) at 540 nm. The optical density (OD) was calculated using the formula: OD (treated cells) × 100/OD (control cells). The IC_50_ values were calculated using the program Graph Pad Prism® 3.0.

#### 2.4.3 Estimation of LD_50_ from IC_50_ values

Data from the *in vitro* tests were used for estimating the starting dose for acute oral systemic toxicity tests in rodent. The *in vivo* starting dose is an estimated LD_50_ value calculated by inserting the *in vitro* IC_50_ value into a regression formula: log LD_50_ (mM/kg) = 0.439 log IC_50_ (mM) + 0.621 [13], [14], [15]. The value is recalculated to mg/kg and compounds are evaluated in accordance with 4 toxicity categories [16]: category 1: LD_50_ ≤ 5 mg/kg (highly toxic); category 2: 5 < LD_50_ ≤ 50 mg/kg (moderately toxic); category 3: 50 < LD_50_ ≤ 300 mg/kg (slightly toxic); category 4: 300 < LD_50_ ≤ 2 000 mg/kg (practically non-toxic).

### 2.5 Self-assembling properties by dynamic light scattering measurements

Compound samples for characterisation with dynamic light scattering (DLS) were prepared by thin-film hydration method in an aqueous solution at a concentration of 0.05 mM. A certain amount of compound was weighted in a round-bottom flask and dissolved in chloroform; then, the organic solvent was removed *in vacuo*, and the residue was dried in high vacuo for 2 h. Deionised water was added and samples were prepared by sonication using a bath-type sonicator (Cole Parmer Ultrasonic Cleaner 8891CPX). Samples were sonicated for 60 min at 50 °C.

The DLS measurements of the nanoparticles in an aqueous solution were carried out on a Zetasizer Nano ZSP (Malvern Panalytical Ltd.) instrument with Malvern Instruments Ltd. Software 7.12, using the following specifications – medium: water; refractive index: 1.33; viscosity: 0.8872 cP; temperature: 25 °C; dielectric constant: 78.5; nanoparticles: liposomes; refractive index of materials: 1.60; detection angle: 173°; wavelength: 633 nm. Data were analysed using the multimodal number distribution software that was included with the instrument.

### 2.6 Statistical analysis

Results are expressed as mean ± standard deviation (SD). All of the biological experiments were performed six times and self-assembling properties by dynamic light scattering measurements three times.

## Declaration of Competing Interest

The authors declare that they have no known competing financial interests or personal relationships which have, or could be perceived to have, influenced the work reported in this article.

## Appendix Supplementary materials

Image, application 1

Image, application 2
