# Supplementary material for: Data for the cytotoxicity, self-assembling properties and synthesis of 4-pyridinium-1,4-dihydropyridines
Source: Data Brief. 2020 Nov 19;33:106545. doi: 10.1016/j.dib.2020.106545 (PMC7701313; doi:10.1016/j.dib.2020.106545)
Supplement: Supplementary file 1 [file mmc1.docx]

Table 1. Cytotoxicity and calculated basal toxicity of 4-pyridinium-1,4-dihydropyridines **3**–**6**

| **Comp.** | **R** | **R’** | **X^-^** | **HT-1080** | | **MH-22A** | | **NIH3T3** | |
| --- | --- | --- | --- | --- | --- | --- | --- | --- | --- |
|  |  |  |  | **IC_50_**  **(CV)**  **µg/mL** | **IC_50_**  **(MTT)**  **µg/mL** | **IC_50_**  **(CV)**  **µg/mL** | **IC_50_**  **(MTT)**  **µg/mL** | **IC_50_**  **(NR)**  **µg/mL** | **LD_50_**  **mg/kg** |
| **3** | C_16_H_33_ | CH_2_CONH_2_ | I | 70±11 | 26±5 | 100±16 | 32±8 | 16±2 | 638 |
| **4** | C_16_H_33_ | CH_2_COOC_2_H_5_ | Br | * | * | * | * | * | >2000 |
| **5** | (CH_2_)_2_OCOC_15_H_31_ | CH_3_ | I | 12±2 | 20±8 | 10±2 | 12±3 | 40±8 | 1020 |
| **6** | C_2_H_5_ | C_16_H_33_ | Br | <<1 | <<1 | <<1 | <<1 | 4±0.4 | 295±45 |

* – not detected
