# Supplementary material for: Data for the cytotoxicity, self-assembling properties and synthesis of 4-pyridinium-1,4-dihydropyridines
Source: Data Brief. 2020 Nov 19;33:106545. doi: 10.1016/j.dib.2020.106545 (PMC7701313; doi:10.1016/j.dib.2020.106545)
Supplement: Supplementary file 2 [file mmc2.docx]

Table 2. Values of average diameter (D_av_), zeta-potential (zeta-pot.) and polydispersity index (PDI) of nanoparticles formed by 4-pyridinium-1,4-DHP derivatives **3**–**6** obtained by dynamic light scattering (DLS) measurements. The average diameter (D_av_) depicts the average hydrodynamic diameter of nanoparticles in the tested sample; the PDI value describes polydispersity of the sample; the zeta-potential gives information about the surface charge of nanoparticles.

| **Comp.** | **D_av_** | **PDI** | **Zeta-pot.** |
| --- | --- | --- | --- |
| **3** | * | 1 | * |
| **4** | * | 1 | * |
| **5** | 88±3 | 0.346±0.034 | 35.1±3.4 |
| **6** | 585±20 | 0.334±0.153 | 0.39±0.54 |

* – not detected
